# Supplementary material for: Causal Relationship between Aging and Anorexia Nervosa: A White-Matter-Microstructure-Mediated Mendelian Randomization Analysis
Source: Biomedicines. 2024 Aug 16;12(8):1874. doi: 10.3390/biomedicines12081874 (PMC11351342; doi:10.3390/biomedicines12081874)
Supplement: Supplementary file 1 [file biomedicines-12-01874-s001.zip › Supplementary Figure/Figure S8/Leaveoneout plot of MR analysis for TL to ubm-b-2068.pdf]

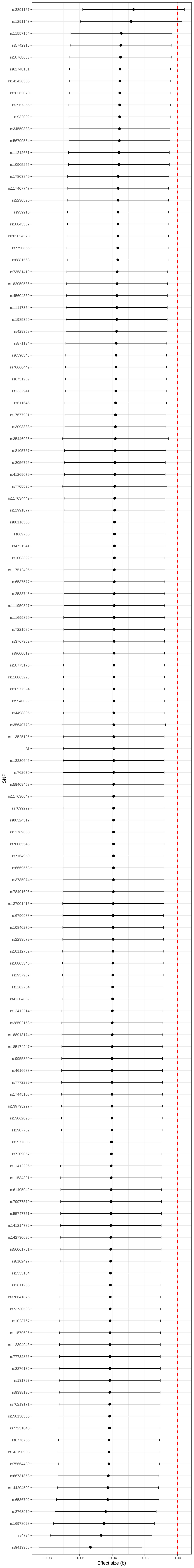
SNP
rs3891167
rs1291143
rs11557154
rs5742915
rs10768683
rs61748181
rs142426306
rs28363070
rs2967355
rs932002
rs34550383
rs56799554
rs11212631
rs10905255
rs17803849
rs117407747
rs2230590
rs939916
rs10845387
rs202034370
rs7790856
rs6881568
rs73581419
rs182059586
rs45604339
rs11117354
rs1985369
rs429358
rs871134
rs6590343
rs76666449
rs6751209
rs1332941
rs611646
rs17677991
rs3093888
rs35446936
rs8105767
rs2056726
rs41269079
rs7705526
rs117034449
rs11991877
rs80116508
rs869785
rs4731541
rs1003322
rs117512405
rs6587577
rs2538745
rs111950327
rs11699829
rs7221585
rs3767952
rs9600019
rs10773176
rs116863223
rs28577594
rs9940099
rs4498805
rs35640778
rs113525195
All
rs13230646
rs762679
rs59409453
rs117630647
rs7099229
rs80324517
rs11769630
rs76065543
rs7164950
rs6669563
rs3785074
rs78491606
rs137901416
rs6790988
rs10840270
rs2293579
rs10112752
rs10805346
rs1957937
rs2282764
rs41304832
rs12412214
rs28502153
rs188918174
rs185174247
rs9955360
rs4616688
rs7772289
rs17445108
rs139795227
rs13062095
rs1907702
rs2977608
rs7209057
rs11412296
rs11584821
rs61405042
rs79977579
rs55747751
rs141214782
rs142730696
rs56061761
rs8102497
rs2555104
rs1611236
rs376641875
rs73730598
rs1023767
rs11579626
rs112394943
rs77732866
rs2276182
rs131797
rs9398196
rs76219171
rs150150565
rs77231040
rs6776756
rs143190905
rs75664430
rs66731853
rs144204502
rs6536702
rs2763979
rs16978028
rs4724
rs9419958
−0.08
−0.06
−0.04
−0.02
0.00
Effect size (b)
